# Antibody Response to the Coronavirus Disease 2019 Ad26.COV2.S Vaccine Among Maintenance Dialysis Patients

**DOI:** 10.1016/j.xkme.2022.100409

**Published:** 2022-01-13

**Authors:** Linda H. Ficociello, Joanna Willetts, Claudy Mullon, Chance Mysayphonh, Ines A. Dahne-Steuber, Curtis Johnson, Melanie Pollan, Sandra Alexander, Jeffrey G. Mulhern, Robert J. Kossmann, Michael S. Anger, Jeffrey L. Hymes

**Affiliations:** 1 Fresenius Medical Care, Global Medical Office, Medical Department, Fresenius Medical Care Renal Therapies Group, Waltham, MA; 2 Medical Department, Fresenius Medical Care Renal Therapies Group, Waltham, MA; 3 Spectra Laboratories, Rockleigh, NJ; 4 Spectra Laboratories, Milpitas, CA; 5 Siemens Healthcare Diagnostics Inc, Tarrytown, NY; 6 Fresenius Medical Care North America, Waltham; 7 Baystate Medical Center, Springfield, MA

To the Editor:

This quality improvement project aimed to determine whether dialysis patients on various dialysis modalities, vaccinated at either dialysis clinics or in the community, differed in their antibody response to the coronavirus disease 2019 (COVID-19) Ad26.COV2.S vaccine. All patients vaccinated with Ad26.COV2.S at 20 dialysis clinics, selected to maximize patients with various dialysis modalities and vaccination settings, were eligible. The antibody response was measured in remnant blood samples from routine laboratory tests performed between July and August 2021. All patients allowed the use of their remnant blood samples collected for routine care for research purposes as a part of the consent form signed upon receiving treatment. As such, no additional study-specific informed consent or institutional review board approval was required. The average time between vaccination and sample draw was 95 ± 12 days. The response was assessed using a semiquantitative chemiluminescent assay for immunoglobulin G directed at the receptor binding domain of the S1 subunit of the severe acute respiratory syndrome coronavirus 2 spike antigen (ADVIA Centaur XP/XPT sCOVG; Siemens Healthcare Diagnostics Inc). The index range was 0.5-750, and indices of >1 were considered reactive.^1^ Although a relationship between the index value and immunity has not been defined, indices of >7 meet the Food and Drug Administration requirement of an acceptable level of a neutralizing titer.^2^^,^^3^

Patients with available measurements (n=839) were divided into in-center hemodialysis patients vaccinated at dialysis clinics (G-HD_clinic_), in-center hemodialysis patients vaccinated in communities (G-HD_community_), peritoneal dialysis patients (G-PD), and home hemodialysis patients (G-HHD). On average, the patients on home modalities were younger (57 and 51 years for G-PD and G-HHD, respectively) than the in-center patients (61 and 66 years for G-HD_clinic_ and G-HD_community_, respectively). Furthermore, the G-HD_community_ patients had higher rates of diabetes, hypertension, and catheter use than those in the other groups. The patients were stratified based on COVID-19 diagnosis in their electronic medical record before antibody measurement. Because COVID-19 history was determined based on electronic medical record documentation, some patients with a positive COVID-19 history might have been misclassified as negative, leading to a higher antibody response observed among those with no prior COVID-19 history.

**Figure 1:**
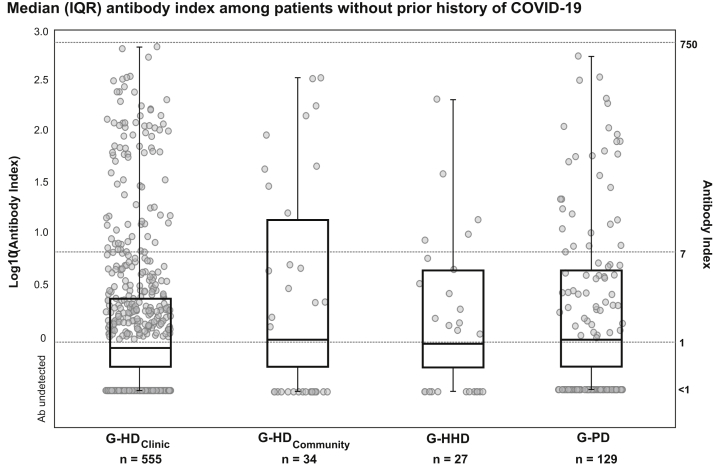
Antibody index levels (along with categorical responses) in dialysis patients without a prior coronavirus disease 2019 history vaccinated with Ad26.COV2.S by group. The horizontal dotted lines are drawn at antibody indexes 1 and 7 to indicate detected and adequate antibody responses and 750 to indicate the maximum detected level. The median (IQR) antibody index of patients without a prior history of COVID-19 was 0.9 (0.5-2.7), 1.2 (0.5-15.9), 1.0 (0.5-4.8), and 1.2 (0.5-4.8) for the G-HD_clinic_, G-HD_community_, G-PD, and G-HHD groups, respectively. Abbreviations: G-HD_clinic_, in-center hemodialysis patients vaccinated at the dialysis clinic; G-HD_community_, in-center hemodialysis patients vaccinated in the community; G-HHD, home hemodialysis patients; G-PD, peritoneal dialysis patients; IQR, interquartile range.

**Figure 2:**
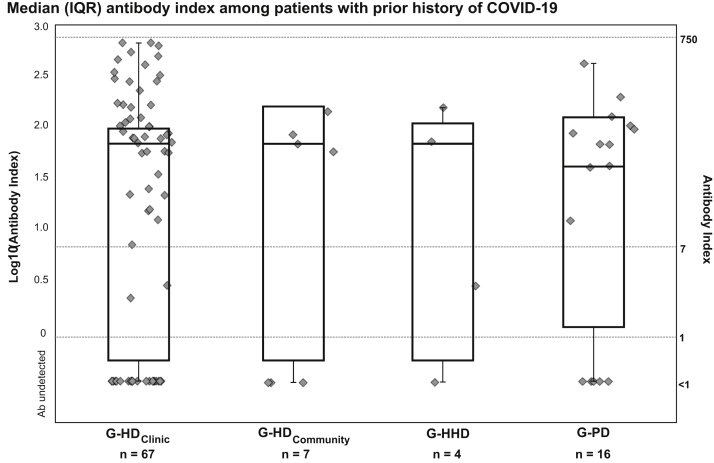
Antibody index levels (along with categorical responses) in dialysis patients with a prior COVID-19 history vaccinated with Ad26.COV2.S by group. The horizontal dotted lines are drawn at antibody index 1 and 7 to indicate detected and adequate antibody response and 750 to indicate the maximum detected level. The median (IQR) antibody index of patients with a prior history of COVID-19 was 61.3 (0.5-90.9), 59.5 (0.5-163.8), 65.2 (0.5-121.3), and 43.7 (1.7-135.2) for the G-HD_clinic_, G-HD_community_, G-PD, and G-HHD groups, respectively. Abbreviations: G-HD_clinic_, in-center hemodialysis patients vaccinated at the dialysis clinic; G-HD_community_, in-center hemodialysis patients vaccinated in the community; G-HHD, home hemodialysis patients; G-PD, peritoneal dialysis patients; IQR, interquartile range.

The antibody levels by group are presented for patients without and with a COVID-19 history in Figs 1 and 2, respectively. Among patients without a COVID-19 history, 53% (294/555), 50% (17/34), 50% (64/129), and 44% (12/27) had an antibody index of <1; 33% (183/555), 24% (8/34), 31% (40/129), and 37% (10/27) had an index of 1-7; and 14% (78/555), 26% (9/34), 19% (25/129), and 19% (5/27) had an index of >7 for the G-HD_clinic_, G-HD_community_, G-PD, and G-HHD groups, respectively. Among patients with a positive COVID-19 history, 31% (21/67), 43% (3/7), 31% (5/16), and 25% (1/4) had an antibody index of <1; 4% (3/67), 0% (0/7), 0% (0/16), and 25% (1/4) had an index of 1-7; and 64% (43/67), 57% (4/7), 69% (11/16), and 50% (2/4) had an index of >7 for the G-HD_clinic_, G-HD_community_, G-PD, and G-HHD groups, respectively. Using a multivariable analysis of variance, we found no difference in the antibody index in terms of the modality (*P* = 0.73) or vaccine administration location (*P* = 0.76); however, there was a difference in terms of prior COVID-19 history (*P* < 0.001; the least-squared mean for a positive COVID-19 history was 111.6 index and for no COVID-19 history was 16.3 index controlling for location and modality).

Patients vaccinated with Ad26.COV2.S had an attenuated antibody response regardless of the modality or administration location. A prior COVID-19 history, not the modality or vaccination setting, had the strongest association with response. Approximately 50% of patients without a COVID-19 history had an unreactive antibody response (an index of <1) across the groups. A recent publication showed a similar attenuated antibody response to the Ad26.COV2.S vaccine in dialysis patients vaccinated at 2 dialysis clinics, where after an average follow-up of 52 days, 62% of patients had an undetected antibody response (an index of <1).^4^ A report by Hsu et al^5^ showed that 63% of patients vaccinated with Ad26.COV2.S had an undetected antibody response; however they found that only 7% of patients vaccinated with messenger RNA vaccines had an undetected antibody response.

Heparin is used in hemodialysis to prevent clotting, and in vitro studies have shown that adenovirus vectors using the clusters of differentiation 46-dependent pathway and heparin sulfate proteoglycans cellular entry pathway are inhibited by heparin.^6^ However, it is unknown whether heparin exposure in dialysis patients at the time of vaccination affects antibody response.

We can assess the potential of heparin administration and its proximity to the timing of vaccination across the modalities. Among the G-HD_clinic_ patients, 99% were treated with dialysis on the same day as vaccination, and 85% had heparin administration documented. Although the exact timing of vaccination in relation to heparin administration was unknown, we may assume that this group had the highest degree of potential heparin exposure compared with the other groups. The G-HD_community_ or G-HHD patients may or may not have been treated with dialysis or heparin on the same day as vaccination (eg, 20% of the G-HD_community_ patients had the documentation of heparin on the same day). The group with the least potential exposure to heparin would be the G-PD patients, who would not be expected to receive heparin. We observed no difference in the antibody response across the modalities or vaccination settings. These results support the continuation of vaccination programs at dialysis clinics.

In summary, most dialysis patients vaccinated with Ad26.COV2.S without a previous history of COVID-19 had an undetectable (52%) or inadequate (33%) antibody index. This finding differs from those of other reports of messenger RNA vaccines showing a high response rate among dialysis patients.^7^ The Centers for Disease Control and Prevention recommends booster shots of any authorized COVID-19 vaccine >2 months after Ad26.COV2.S, although boosters of Ad26.COV2.S are not recommended for those who develop thrombosis with thrombocytopenia syndrome after Ad26.COV2.S vaccination.^8^ Fresenius Kidney Care dialysis clinics are currently administering messenger RNA booster vaccines to its patients, although the patients may receive Ad26.COV2.S within the community.
